# DENdb: database of integrated human enhancers

**DOI:** 10.1093/database/bav085

**Published:** 2015-09-05

**Authors:** Haitham Ashoor, Dimitrios Kleftogiannis, Aleksandar Radovanovic, Vladimir B. Bajic

**Affiliations:** ^1^Computational Bioscience Research Center (CBRC), Computer, Electrical and Mathematical Sciences and Engineering Division (CEMSE) and; ^2^Computer, Electrical and Mathematical Sciences and Engineering Division (CEMSE), King Abdullah University of Science and Technology (KAUST), Thuwal 23955-6900, Saudi Arabia

## Abstract

Enhancers are *cis*-acting DNA regulatory regions that play a key role in distal control of transcriptional activities. Identification of enhancers, coupled with a comprehensive functional analysis of their properties, could improve our understanding of complex gene transcription mechanisms and gene regulation processes in general. We developed DENdb, a centralized on-line repository of predicted enhancers derived from multiple human cell-lines. DENdb integrates enhancers predicted by five different methods generating an enriched catalogue of putative enhancers for each of the analysed cell-lines. DENdb provides information about the overlap of enhancers with DNase I hypersensitive regions, ChIP-seq regions of a number of transcription factors and transcription factor binding motifs, means to explore enhancer interactions with DNA using several chromatin interaction assays and enhancer neighbouring genes. DENdb is designed as a relational database that facilitates fast and efficient searching, browsing and visualization of information.

**Database URL:** http://www.cbrc.kaust.edu.sa/dendb/

## Introduction

Deciphering complex gene regulatory mechanisms requires consideration of interactions between DNA regulatory modules (1). These DNA regulatory modules are composed of regulatory elements that reside in the so-called DNA regulatory regions. These regions can act proximal or distal relative to the genes they affect (2). The proximal regions are located close to the transcription start sites (TSSs) of genes. Promoters are a group of better-characterized proximal regions (3). In contrast to the proximal regions, distal regions are not located close to the genes they affect, but are positioned further away and can even reside at different chromosomes. Enhancers represent one of the better-characterized distal regulatory regions. They are defined as *cis*-acting DNA regions that increase the transcriptional output of a subset of genes (4). Providing a more precise definition of enhancers is not easy as enhancers manifest distinct properties across different tissues and can act bi- directionally with respect to their target genes. Moreover, enhancer regions play a key role in tissue-specific gene expression and can have different roles depending on different cellular conditions (i.e. can be active or can assume non-enhancer function in some cell types) (5).

Recently, genome regulation consortia, such as ENCODE (6), NIH Epigenome Roadmap (7) and FANTOM (8, 9), produced massive amounts of ChIP-seq (10) and CAGE (11) data to help improving our understanding of the gene regulation process at genome-wide scale. This increase in data volume required a systematized way to identify variety of regulatory regions in DNA and thus the development of computational methods for identifying such regions, including enhancers. Examples of methods for enhancer identification are: ChromHMM (12), Segway (13), RFECS (14), DEEP (15), CSI-ANN (16), EnhancerFinder (17), kmer-SVM (18, 19) and ChroModule (20). Characteristics of the methods used in DENdb are summarized in Supplementary Table S1.

Although these methods increased the set of computationally predicted enhancers in a large number of available cell-lines, this information could be misleading and cannot be easily utilized due to specific technical limitations that frequently prevent or restrict the usage and the analysis of the data. The most important obstacles are listed below:

- The available computationally predicted enhancers generated by different prediction methods differ significantly from method to method; this would benefit from integration of these predicted enhancers into a centralized repository based on large-scale archiving that would enable systematic searching, browsing, comparing, combining and visualizing relevant information.
- As different methods are trained on some cell-lines and tested on others, it makes sense to combine the available predictions and generate cell-specific annotation maps of enhancers based on different levels of confidence and overlaps between predictions; however, up to now, to the best of our knowledge, the only available integrated annotation of enhancers is based on ChromHMM and Segway (21).
- Current enhancer databases (8, 22, 23) do not provide utilities to analyse archived enhancers in a number of important aspects that would facilitate exploration of gene regulation mechanisms, such as (i) overlaps of enhancers with transcription factor (TF) binding motifs, (ii) overlaps of enhancers with relevant experimental data such as chromatin accessibility as captured by DNase I hypersensitivity sites (DHSs), (iii) linking enhancers to closest genes (that in the first approximation could be considered as candidate target genes of an enhancer) and (iv) associations of enhancers with chromatin conformation based on experimental information (i.e. 3C or ChIA-PET).

Such utilities will help obtaining information that can describe more completely functional context of enhancer activities in different cell-lines and thus help to increase our understanding of gene regulation processes under different cellular conditions.

With all these issues in mind, we introduce DENdb, Dragon Enhancers database. DENdb is a user-friendly online repository of enhancers predicted by different methods in various human cell-lines. Combining these data, DENdb generates an integrated comprehensive catalogue of enhancers for 15 human cell-lines. Beyond the integrated set of enhancers, DENdb integrates other sources of information to help researchers explore functional context of possible enhancer activities. These sources include TF ChIP-seq data from ENCODE and TF binding motifs based on HOCOMOCO (24) TF binding sites (TFBS) models, DHS information from ENCODE experiments and eRNA (enhancer RNA) expression values from FANTOM5 (8). Finally, DENdb attempts to link enhancers to their target genes by integrating chromatin interaction assays and defining the closest gene for each enhancer. DENdb is freely available at http://www.cbrc.kaust.edu.sa/dendb/.

## Materials and methods

### Enhancer sources

DENdb enhancer collection contains computationally predicted regions obtained by five different methods, namely: CSI-ANN, Segway, ChromHMM, RFECS and the ENCODE integrative annotation. To obtain these enhancer predictions, we focused mainly on ENCODE ChIP-seq histone modifications data.

CSI-ANN feeds a linear combination of histone modifications information at a certain window to a time-delay neural network in order to predict enhancers. CSI-ANN model used in DENdb is based on P300 binding sites distal to TSS as determined by CD14^+^ T cells (25). CSI-ANN model was trained on data from three histone modifications (H3K4me1, H3K4me2 and H3K4me3) obtained from Wang *et al.* (26). RFECS uses a multivariate random forest to capture chromatin signatures at enhancer regions. RFECS uses regions with P300 binding sites distal to TSS and overlapping with DHS sites from H1 and IMR90 cell-lines as their enhancer regions. RFECS model is trained using three histone modification marks H4K4me1, H3K4me2 and H3K4me3. ChromHMM uses a semi-automated approach to segment the genome. Initially, it uses hidden Markov model to segment the genome into multiple clusters. Later on, domain experts have annotated each cluster manually. It uses histone modifications ChIP-seq data to perform this operation. ChromHMM builds a single model by cascading data from nine different cell-lines (27). Segway uses a similar semi-automated approach. However, it utilizes dynamic Bayesian networks to construct genome segments. It uses 1% of the genome to construct its model. Also, it constructs a single model for each cell-line. To capture characteristics from both genome segmentations, an integrative annotation (21) is used based on ChromHMM and Segway annotations as well as a set of other experimental data. The integration process was done manually for both segmentations.

In DENdb, we used original CSI-ANN and RFECS models to predict enhancers for all the ENCODE cell-lines that contain histone modification marks required as input for these programs. In addition, we extracted enhancer’s related states from the three segmentation models. Supplementary Table S2 shows the links for enhancer sources used in DENdb.

In DENdb, based on the number of methods used for predicting enhancers in each cell-line, cell-lines are categorized into two tiers. Tier 1 includes cell-lines that have predictions from all five methods, whereas Tier 2 includes cell-lines that have predictions from two (CSI-ANN and RFECS) methods. Supplementary Table S3 shows the original enhancer count from each method per cell-line.

### Integrating enhancer predictions

Initially, we binned the genome into 50-bp non- overlapping intervals. Then, we mapped enhancer predictions from all different methods to obtain enhancers super track that has predictions from all methods for each cell-line. We grouped regions that contain one prediction or more into our integrated enhancers. For each region, we define the support by maximum number of methods whose predictions cover at least *M* bins. In the current implementation of DENdb, we set *M* to be 2 (100 bp).

### DHS data

In addition to enhancers, DENdb integrates DHS information obtained from http://ftp.ebi.ac.uk/pub/databases/ensembl/encode/integration_data_jan2011/byDataType/openchrom/jan2011/fdrPeaks/. DHS data can be used to increase the confidence of enhancer predictions (28).

### Information for TF binding

In DENdb, we integrate two types of TF binding information: (i) TF binding regions based on ChIP-seq data and (ii) predicted TF binding motifs using HOCOMOCO TFBS models.

For TF binding region from ChIP-seq data, we integrated all uniform ChIP-seq peaks for TFs produced by ENCODE consortium that overlaps with DENdb-integrated enhancers.

We mapped 426 (A–D quality) models from HOCOMOCO database to our integrated enhancers. We used FIMO (29) to map position weight matrix derived from binding sites for each TF to all enhancers. We set the false discovery rate for accepting predicted binding motif occurrence to 0.1.

### Defining the closest genes

We used ClosestBed functionality from Bedtools (30) to associate each enhancer with its closest gene from Refseq (release 68) (31). In DENdb, for each enhancer we report its closest gene and its distance from the gene.

### Chromatin interaction information

DENdb integrates chromatin interaction information from different high throughput assays namely 3C, 4C, 5C and ChIA-PET obtained from 4DGenome database (32). We used Bedtools (30) to associate, enhancers with existing interacting DNA regions. If available, we also reported known genes that lie within the interaction pairs regions.

### FANTOM5 enhancer expression

FANTOM5 enhancers are obtained from Atlas of transcribed enhancers (8). We reported cell-specific expression of all FANTOM5 permissive enhancers that overlap with DENdb-integrated enhancers. Expression values are provided as log_2_ (Tag per Million).

### The implementation

**Figure 1.**
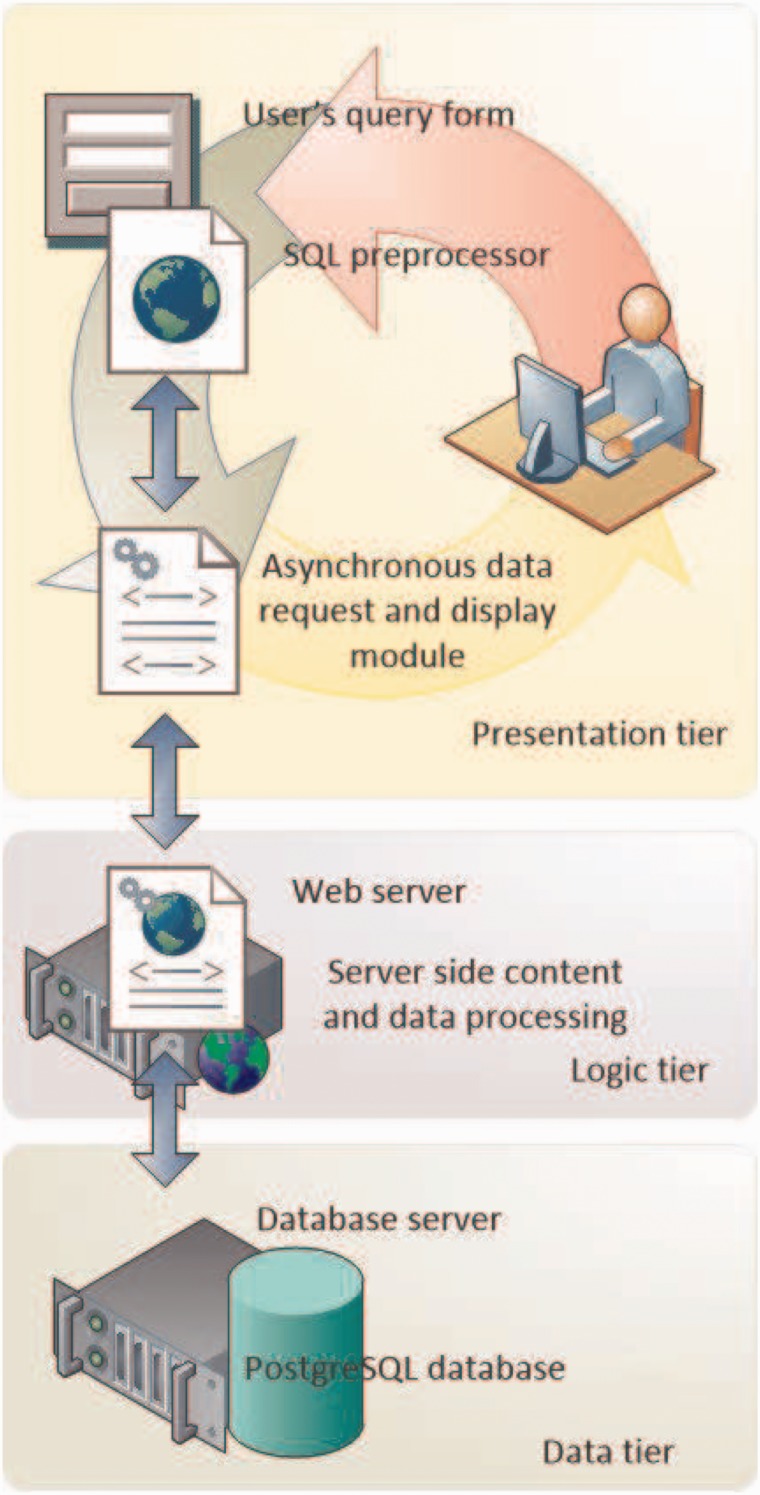
DENdb’s implementation employs the three-tier architecture approach. This includes data, logic and presentation tiers.

DENdb architecture is built around three-tier model shown in Figure 1. This architecture provides scalable, easy to maintain high performing software. The data tier includes PostgreSQL relational database (http://www.postgresql.org/) with PostGIS (http://postgis.refractions.net/) extension to effectively handle integer range queries. The logic tier contains most of the application logics and handles data transfer between data and presentation tiers. It is implemented in PHP scripting language by using object-oriented approach. Presentation tier handles user interaction, requests and display results obtained from the bottom tiers. It is implemented in HTML5/CSS3 and jQuery (http://jquery.com/).

## Results

We developed DENdb, an integrated database of predicted enhancers in 15 human ENCODE cell-lines. The current DENdb implementation contains enhancers predicted by five different methods and integrates regulatory-based information based on the data from five other sources. Table 1 summarizes sources of information used in DENdb compilation and their statistics.

**Table 1.** DENdb data sources statistics

Data type	Number of entries	Number of cell-lines
Integrated enhancers	3 506 396	15
DHS regions	769 947	13
ChIP-seq TFBSs	4 005 967	15
HOCOMOCO predicted TFBSs	94 074 558	15
Chromatin interactions	778 061	6
FANTOM5 CAGE expression	80 291	8

**Figure 2.**
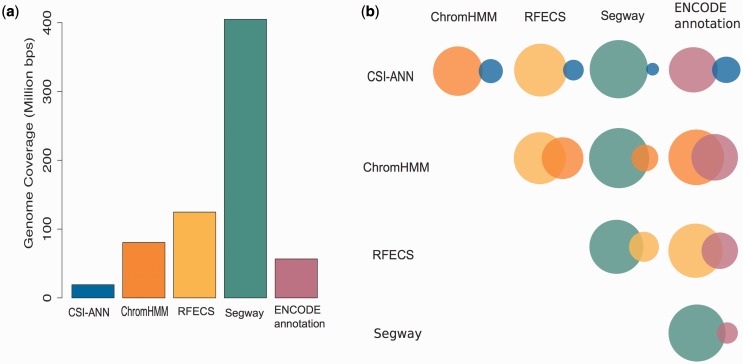
Statistics for H1hesc cell-line enhancers. (**a**) Bar plot represents genome coverage in million base pairs by each method individually. (**b**) Venn diagrams show the pairwise intersection between the predictions of five tools used in DENdb. Size of the circle represents relative proportion of predictions for a method compared to the union of both methods.

As enhancer prediction methods use different definitions of enhancers, as well as different data and techniques to predict enhancers, it is expected that predictions of those methods are different. Figure 2 summarizes individual predictions of enhancers in H1hesc cell-line (similar statistics for other cell-lines can be found in Supplementary Figures S1–S5). One can observe that the five prediction methods used produce highly diverse sets of enhancer predictions. In DENdb, we combine these predictions, report the integrated form of these predictions and provide a support value for each integrated enhancer. Such information can help researchers in exploring richer sets of enhancers than available by the use of individual methods, but also to explore the sets of enhancers with different levels of support (e.g. enhancers supported by, say, three different methods vs. enhancers supported by two methods). In addition to support (confidence level) of enhancers, users can explore their query results using other utilities included in DENdb, such as checking for an enhancer overlap with DHS site, chromatin interaction region, TF binding region or motif for a specific TF.

Enhancers of different cell-lines are characterized by different sets of active TFs that present enrichment of respective TF binding motifs (33). DENdb contains information from 168 of ENCODE TF ChIP-seq data and 426 TFBS models from HOCOMOCO database to select from. For predicted TF binding motifs based on HOCOMOCO models, we report the list of top-five enriched motifs in each cell-line; this information can be found in Supplementary Table S5. A complete list of counts of occurrences of TFs in enhancers per cell-line can be found at Supplementary Table S6.

### Utility and function

**Figure 3.**
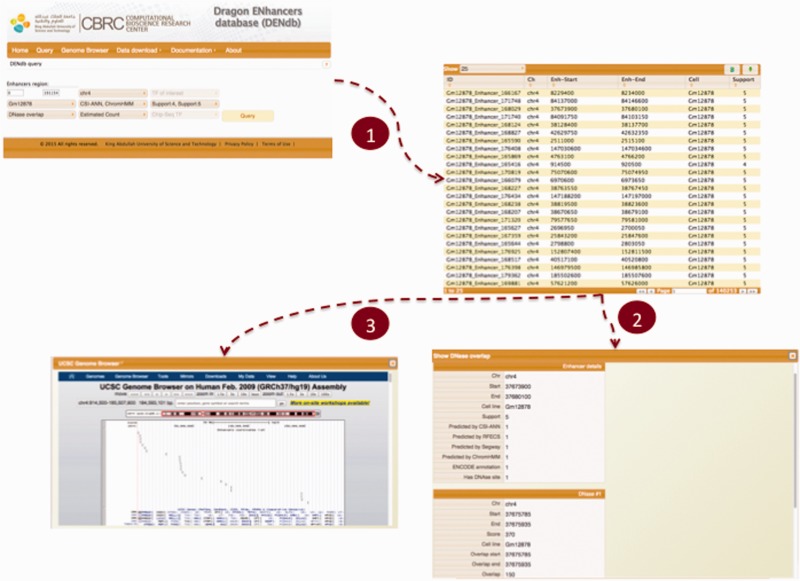
Snapshot of DENdb. An example of querying enhancers that overlap with DHS regions. Queried enhancers are from GM12878 cell-line and from chromosome 4. The query example specifies enhancers having support of 4 or 5 only and being predicted by CSI-ANN and ENCODE ChromHMM. After step 1, results appear in a tabular format. Step 2 shows exploring details of a specific enhancer. Step 3 shows visualizing enhancers in current page in genome browser.

DENdb allows users to perform multiple explorations of data, which span from simple browsing of the database to more customized queries that may include, for example, search for enhancers based on the simultaneous use of many criteria. DENdb queries can be customized by chromosome, coordinates range, cell-line, enhancer support, as well as the method that has generated the enhancer predictions. DENdb allows user to query enhancers that overlap with some genomic features or has a specific property. For example DENdb allows user to explore overlapping of enhancers with DHS region, TF ChIP-seq peaks, prediction of TF binding motifs by HOCOMOCO TFBS models or chromatin interaction region, and can also provide information about eRNA expression and enhancer’s closest gene. For example, the following three criteria may be requested: (i) location on a specific chromosome, (ii) overlap with DHS regions and (iii) support greater than the user defined threshold. Figure 3 shows a snapshot of DENdb showing some of its utilities.

Users can explore each enhancer obtained from any of DENdb queries by inspecting all its basic details, such as cell-line, support, tools predicting this enhancer and coverage by DHS region. In addition, query-specific information are available for each query such as size of overlap with DHS regions actual loci of predicted TF binding motifs, and the source of TF ChIP-seq data.

DENdb query results can be downloaded in the BED format. Also, DENdb provides the means to visualize query results using UCSC genome browser. A user manual for DENdb is available at the DENdb website.

## Discussion

Identifying enhancers are critical starting-points for understanding their functional mechanism and decrypting complex molecular principles that drive cell-specific gene activities. Studying enhancer’s activity across different cell-lines may also provide new insights about different gene expression programs that characterize physiological, as well as pathogenic conditions in cells.

On the other hand, the current enhancer databases (8, 22, 23, 34, 35) have some limitations. For example, VISTA enhancer browser (22) focuses on developmental enhancers only, Atlas of transcribed enhancers (8) provides a limited capacity to explore functional context of enhancer activity, and Cistrome DB (23) contains very limited information about enhancers. There are some other enhancer databases, such as PReMod (35) which contains computationally predicted enhancers based on the conserved regulatory modules and predicted TF binding motifs, and PEDB (34) that contains predicted enhancers based on TSSs and conserved non-coding elements (CNE). However, these databases provide no connection to the new experimental data. There are two unpublished enhancer databases, ZenBase (http://zenbase.genereg.net/) and dbSuper (http://bioinfo.au.tsinghua.edu.cn/dbsuper/). The first one presents enhancers obtained by human/zebrafish comparison of highly CNE (36), whereas the other one contains information about super enhancers for human and mouse (37).

To enhance the capacity of users to analyse the enhancer information, we developed DENdb, a database of computationally predicted human enhancers. DENdb is an on-line archive of enhancer regions obtained by five prediction methods and currently covers 15 different ENCODE cell-lines. The prediction methods we used all rely on ChIP-seq histone modification marks data obtained by ENCODE experiments. DENdb provides users the utility to explore some aspects of gene regulation mechanisms by overlapping enhancer predictions with DHS data and TFBSs. Different subsets of enhancers could be selected based on the level of support from different prediction methods. In addition, DENdb provides possibility for more complex queries about functional context of enhancer activity across cell-lines. These requirements are achieved by focusing on regions that are supported by predictions of multiple methods. Integrating these datasets into a single data repository enables development of a new enhancer annotation based on various methods that have been developed under different assumptions.

The main distinguishing characteristics of DENdb compared with other existing enhancer databases is that it provides (i) annotation of integrated enhancers based on five recent different enhancer prediction methods; and integration of enhancer information with (ii) DHS experimental data; (iii) TF-based ChIP-seq peaks and TF binding motifs; (iv) the transcriptomic data from FANTOM5; and (v) chromatin interaction information from 3C, 4C, 5C and ChIA-pet experimental data. DENdb contains information from 15 ENCODE cell-lines and it provides query system to explore the above-mentioned integrated information about the enhancers.

In future, we plan to further improve DENdb. We plan to integrate single nucleotide polymorphisms information and associate enhancers with specific phenotypes. We also plan to derive set of super enhancers (38) based on set of integrated enhancers. We hope that DENdb will help researchers in gene regulation domain in studying genome-wide human regulatory regions of interest and in decrypting the complex gene transcriptional mechanisms.

## Supplementary Data

Supplementary data are available at *Database* Online.

## Funding

Funding for this study is obtained from King Abdullah University of Science and Technology (KAUST) Research Funds through AEA KAUST-Stanford Round 3 Global Collaborative Research Program, and KAUST Base Research Funds to V.B.B. Funding for open access charge: KAUST Base Research Funds to V.B.B.

*Conflict of interest*. None declared.

## Supplementary Material

Supplementary Data
