# Supplementary material for: DENdb: database of integrated human enhancers
Source: Database (Oxford). 2015 Sep 5;2015:bav085. doi: 10.1093/database/bav085 (PMC4560934; doi:10.1093/database/bav085)
Supplement: Supplementary Data [file supp_2015_bav085_index.html]

DENdb: database of integrated human enhancers — Supplementary Data 

# DENdb: database of integrated human enhancers

## Supplementary Data

files

- Supplementary Data - zip file
