# Supplementary material for: DENdb: database of integrated human enhancers
Source: Database (Oxford). 2015 Sep 5;2015:bav085. doi: 10.1093/database/bav085 (PMC4560934; doi:10.1093/database/bav085)
Supplement: Supplementary Data [file supp_bav085_suppl_data.zip › DENdb_Supplementary_Figures.docx]

**DENdb: database of integrated enhancers in human**

Haitham Ashoor ^1^, Dimitrios Kleftogiannis ^2^, Aleksandar Radovanovic ^1^ and Vladimir B. Bajic ^1,*^

^1^ Computational Bioscience Research Center (CBRC), Computer, Electrical and Mathematical Sciences and Engineering Division (CEMSE), King Abdullah University of Science and Technology (KAUST), Thuwal, 23955-6900,Saudi Arabia

^2^ Computer, Electrical and Mathematical Sciences and Engineering Division (CEMSE), King Abdullah University of Science and Technology (KAUST), Thuwal, 23955-6900, Saudi Arabia.

* Corresponding author

Email of the corresponding author: vladimir.bajic@kaust.edu.sa

**Supplementary Figures**


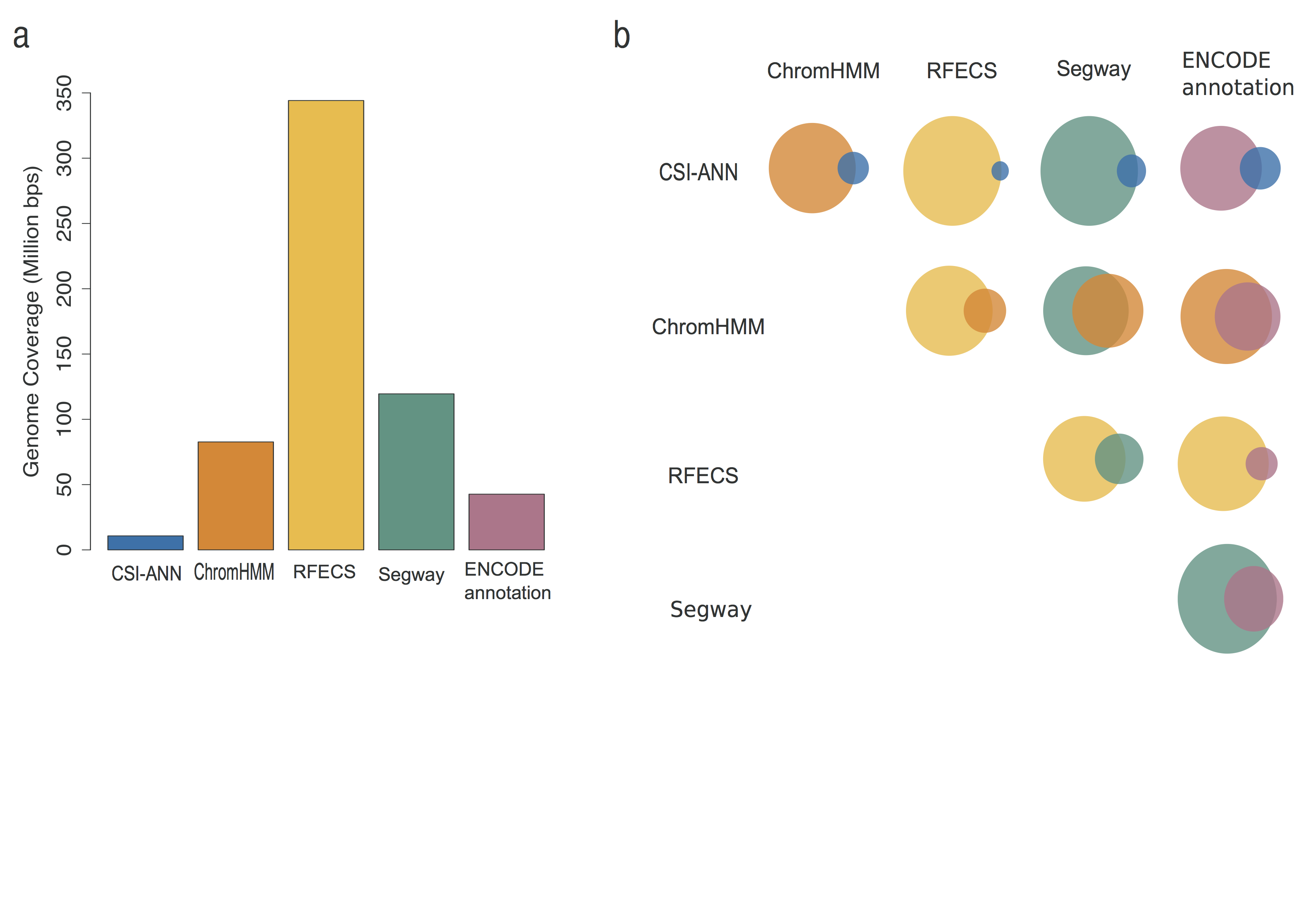
**Figure S1:** Statistics for Gm12878 cell-line enhancers.


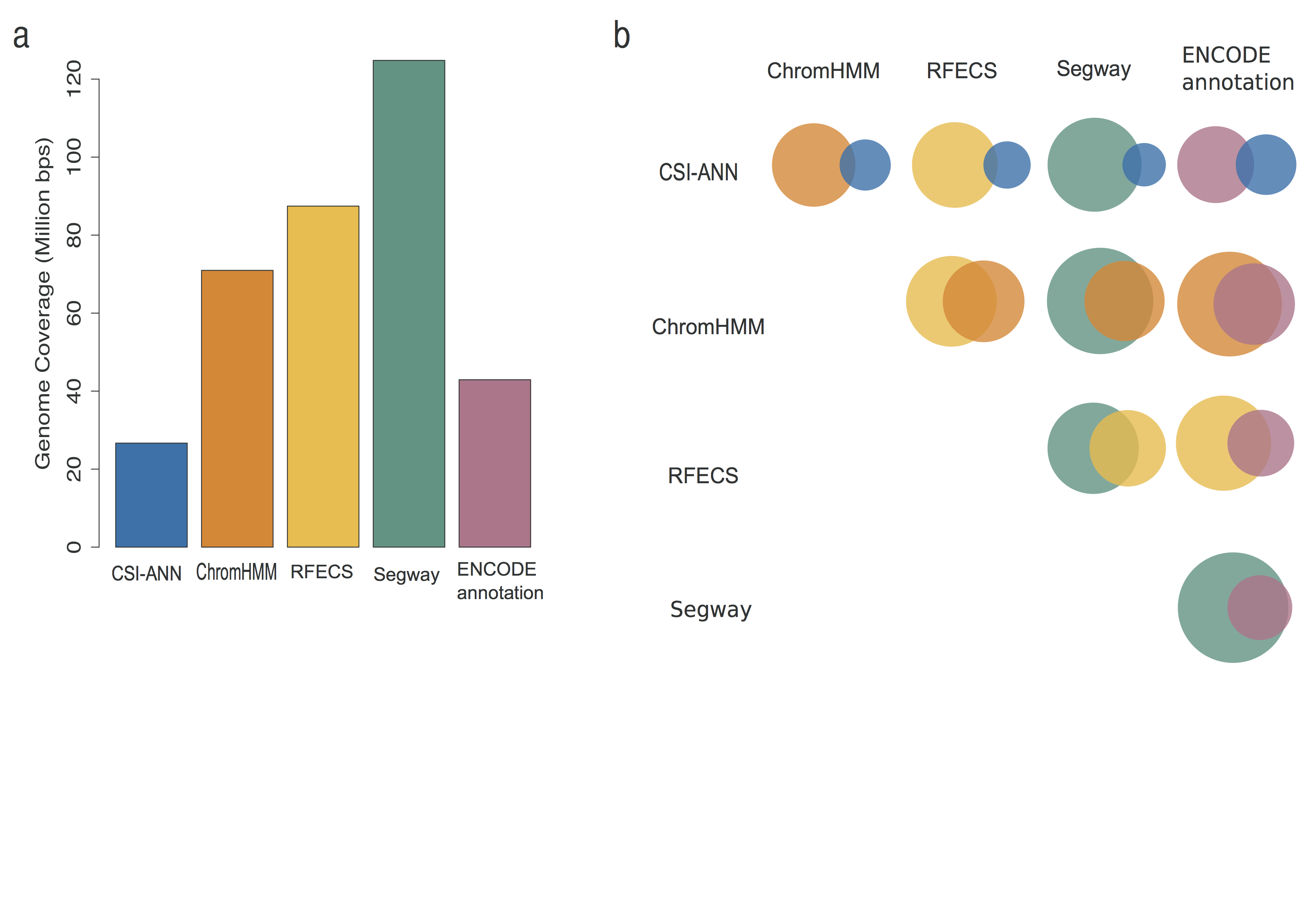
**Figure S2:** Statistics for HelaS3 cell-line enhancers.


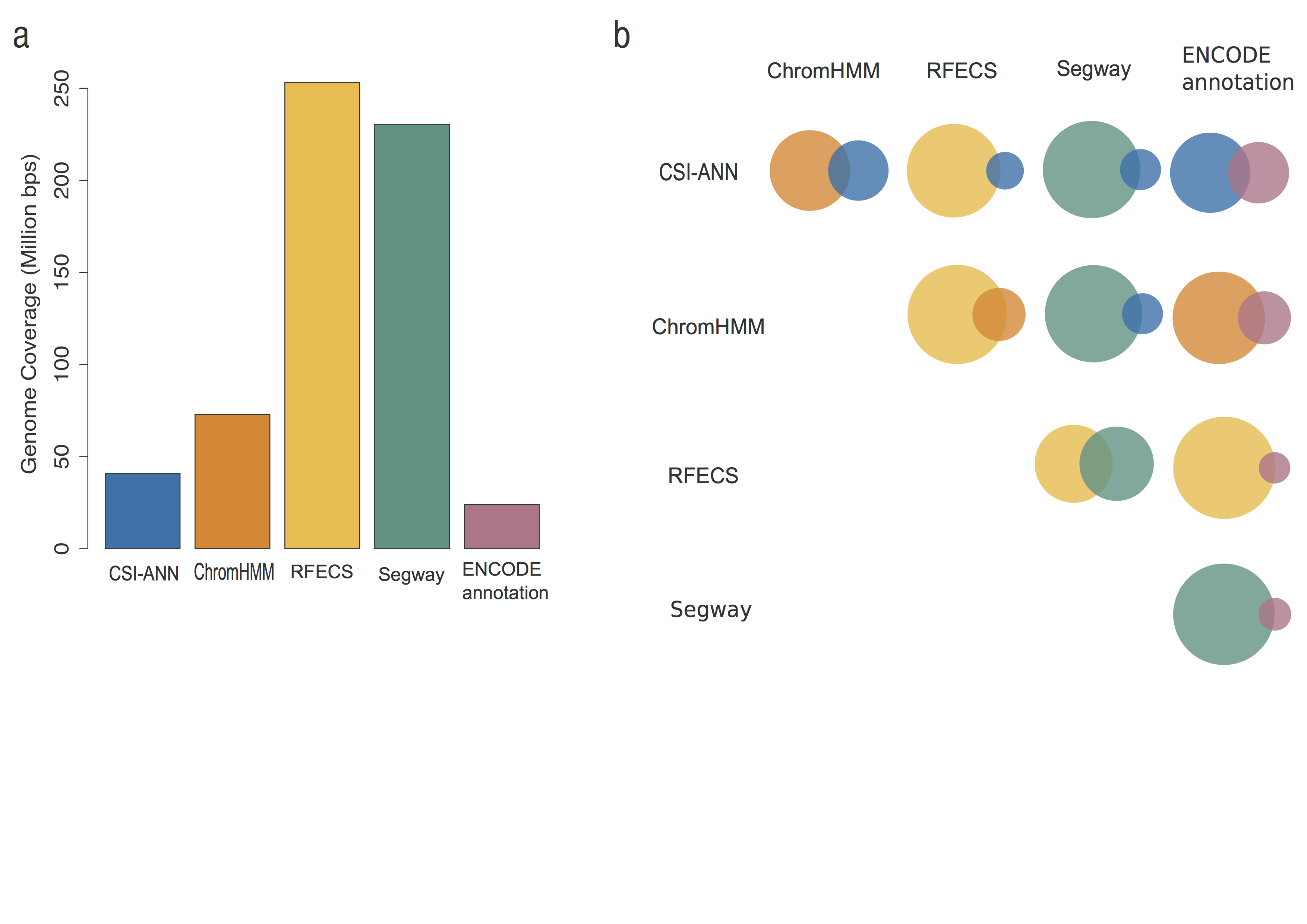
**Figure S3:** Statistics for HepG2 cell-line enhancers.


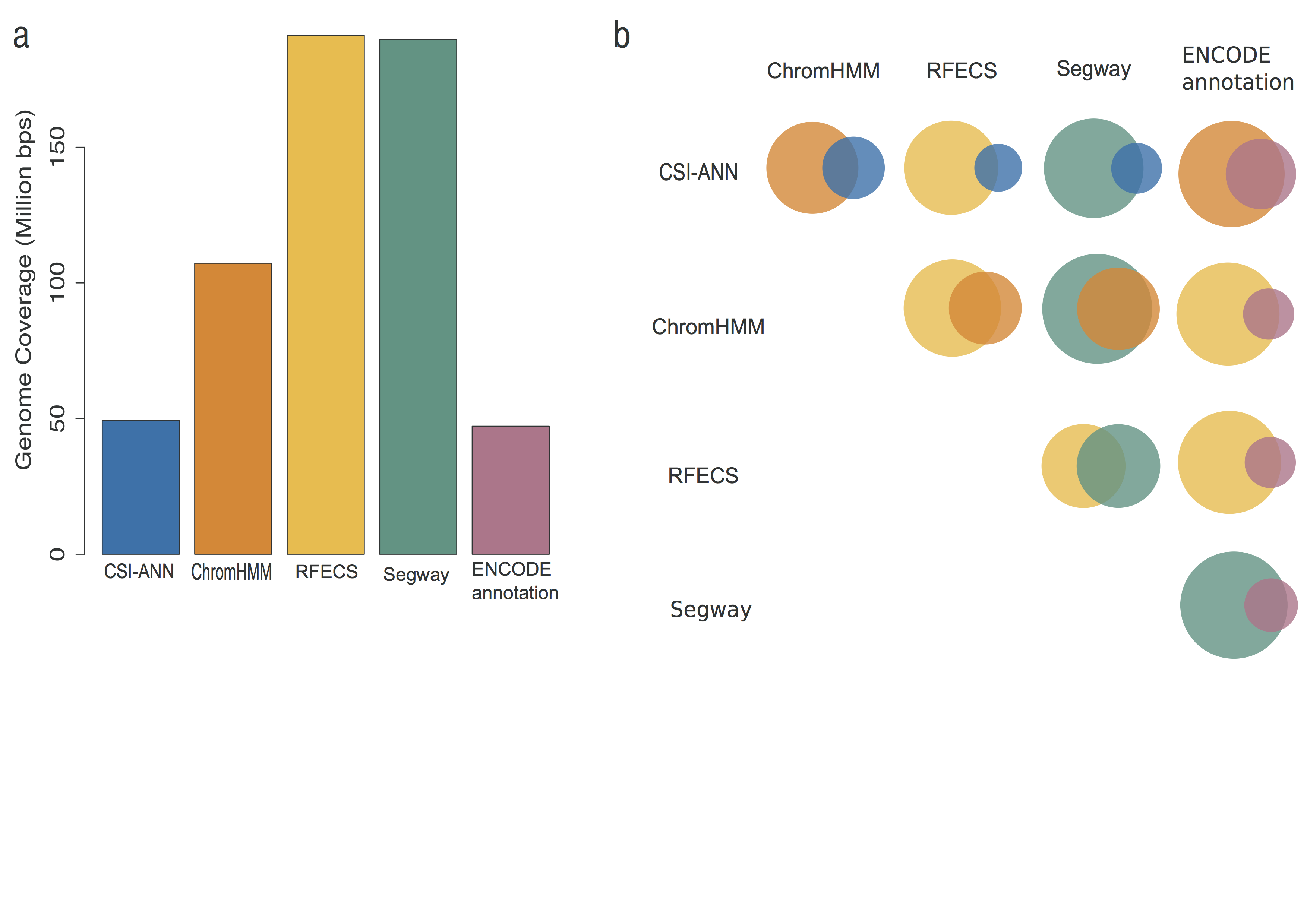
**Figure S4:** Statistics for Huvec cell-line enhancers.


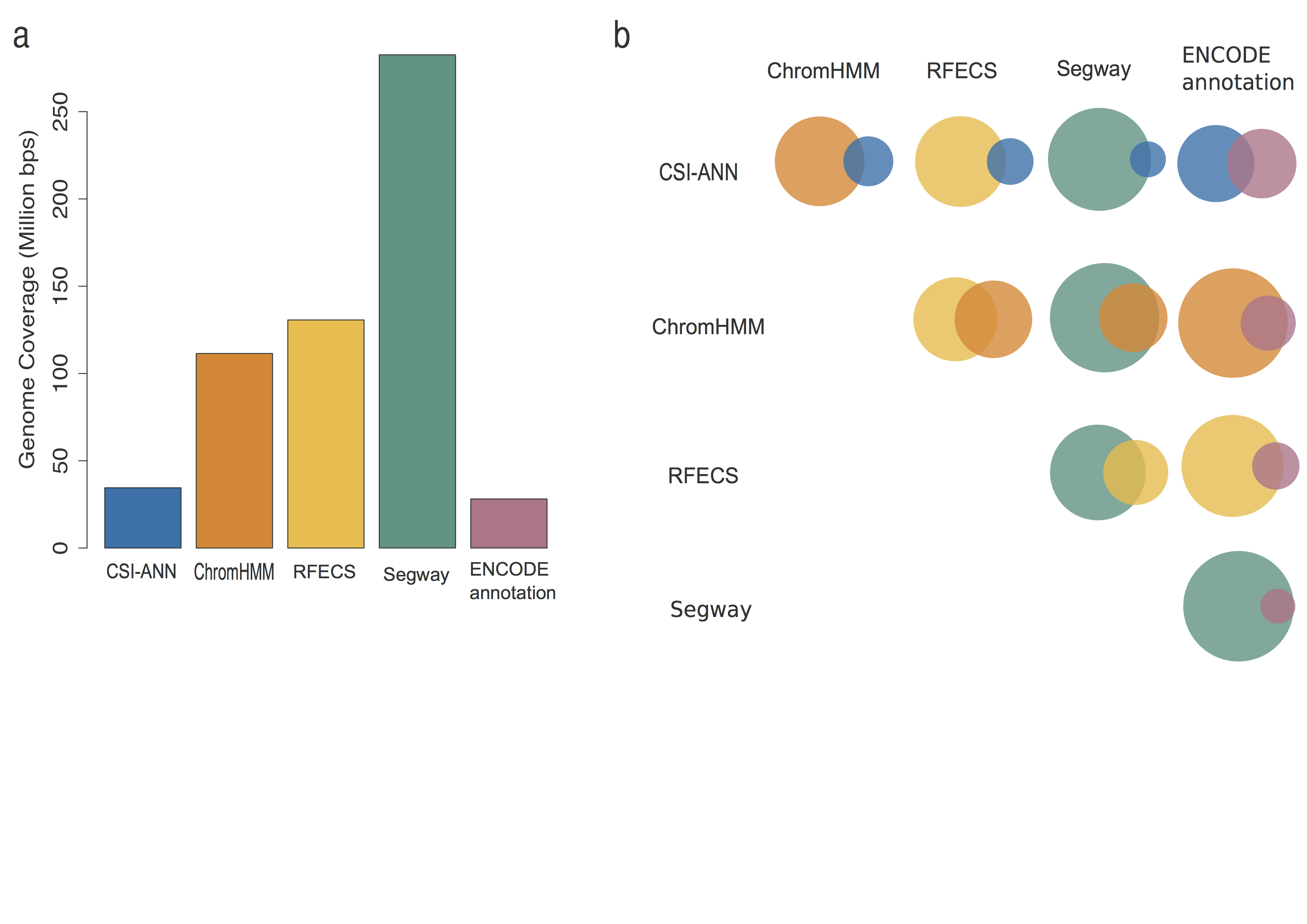
**Figure S5:** Statistics for K562 cell-line enhancers.
