# Supplementary material for: DENdb: database of integrated human enhancers
Source: Database (Oxford). 2015 Sep 5;2015:bav085. doi: 10.1093/database/bav085 (PMC4560934; doi:10.1093/database/bav085)
Supplement: Supplementary Data [file supp_bav085_suppl_data.zip › DENdb_Supplementary_Tables.docx]

**DENdb: database of integrated enhancers in human**

Haitham Ashoor ^1^, Dimitrios Kleftogiannis ^2^, Aleksandar Radovanovic ^1^ and Vladimir B. Bajic ^1,*^

^1^ Computational Bioscience Research Center (CBRC), Computer, Electrical and Mathematical Sciences and Engineering Division (CEMSE), King Abdullah University of Science and Technology (KAUST), Thuwal, 23955-6900,Saudi Arabia

^2^ Computer, Electrical and Mathematical Sciences and Engineering Division (CEMSE), King Abdullah University of Science and Technology (KAUST), Thuwal, 23955-6900, Saudi Arabia.

* Corresponding author

Email of the corresponding author: vladimir.bajic@kaust.edu.sa

**Supplementary Tables**

**Table S1**: Summary of the current methods for enhancer identification

| **Program name** | **Method Description** | **Input data** | **Number of cell-lines** |
| --- | --- | --- | --- |
| ChromHMM | Hidden Markov Models (HMM) and unsupervised clustering of profiles to segment genome in different states including enhancers. | ChromHMM accepts aligned ChIP-seq reads for each histone mark. In DENdb we collected the published annotation for ChromHMM | 6 |
| CSI-ANN | Artificial Neural Network (ANN) approach for identifying enhancers from histone marks | CSI-ANN requires aligned ChIP-seq reads for each histone mark | 15 |
| RFECS | Random Forest (RF) based approach for predicting enhancers from histone marks. | Requires aligned ChIP-seq reads of histone modifications. | 15 |
| Segway | Dynamic Bayesian network for genome segmentation in different states including enhancers | Segway requires aligned ChIP-seq reads for each histone mark. In DENdb we collected the published annotation for Segway | 6 |

**Table S2:** Enhancer sources links

| Enhancer source | Link |
| --- | --- |
| CSI-ANN program | <http://www.healthcare.uiowa.edu/labs/tan/CSIANNWebpage.html> |
| RFECS program | <http://enhancer.ucsd.edu/renlab/RFECS_enhancer_prediction/> |
| ChromHMM segmentations | <http://ftp.ebi.ac.uk/pub/databases/ensembl/encode/integration_data_jan2011/byDataType/segmentations/jan2011/hub/> |
| Segway | <http://ftp.ebi.ac.uk/pub/databases/ensembl/encode/integration_data_jan2011/byDataType/segmentations/jan2011/hub/> |
| ENCODE integrative segmentation | <http://ftp.ebi.ac.uk/pub/databases/ensembl/encode/integration_data_jan2011/byDataType/segmentations/jan2011/hub/> |

**Table S3**: Enhancer statistics for each method per cell line

| **Cell line** | **CSI-ANN** | **ChromHMM** | **Segway** | **RFECS** | **Integrative ENCODE**  **Segmentation** |
| --- | --- | --- | --- | --- | --- |
| Dnd41 | 18,681 | - | - | 43,681 | - |
| Gm12878 | 6,380 | 156,118 | 421,147 | 172,084 | 86312 |
| H1hesc | 9,613 | 172,075 | 2,338,070 | 62,361 | 156,491 |
| HelaS3 | 13,354 | 138,530 | 459,740 | 43,722 | 89,431 |
| HepG2 | 20,473 | 140,959 | 1,054,299 | 126,583 | 56,137 |
| Hmec | 24,066 | - | - | 93,429 | - |
| Hsmm | 22,538 | - | - | 142,076 | - |
| Hsmmt | 23,026 | - | - | 75,800 | - |
| Huvec | 24,740 | 208,081 | 681,931 | 95,614 | 61,958 |
| K562 | 17,309 | 205,137 | 1,237,490 | 65,329 | 75,454 |
| MonoCD14 | 31,162 | - | - | 63,893 | - |
| Nha | 19,553 | - | - | 139,170 | - |
| Nhdfad | 21,343 | - | - | 147,797 | - |
| Nhek | 33,207 | - | - | 99,907 | - |
| Nhlf | 16,326 | - | - | 137,077 | - |

**Table S4:** Total count of enhancers per cell line

| **Cell line** | **Enhancers numbers** |
| --- | --- |
| Dnd41 | 3,674 |
| Gm12878 | 267,944 |
| H1hesc | 1,444,022 |
| HelaS3 | 215,364 |
| HepG2 | 658,668 |
| Hmec | 6,612 |
| Hsmm | 14,593 |
| Hsmmt | 5,807 |
| Huvec | 324,717 |
| K562 | 535,171 |
| MonoCD14 | 5,545 |
| Nha | 4,894 |
| Nhdfad | 10,634 |
| Nhek | 6,222 |
| Nhlf | 8,336 |

**Table S5**: Top five enriched HOCOMOCO TFs in DENdb

| **Cell line** | **Top 5 HOCOMOCO TFs** |
| --- | --- |
| Dnd41 | MAZ_f1, SP4_f1, SP1_f2, SP3_f1, ZBT7B_si |
| Gm12878 | IRF4_si, FOXJ3_si, MAZ_f1, SP3_f1, SP4_f1 |
| H1hesc | PURA_f1, RREB1_si, SP1_f1, PAX5_si, PITX2_si |
| Helas3 | IRF4_si, MAZ_f1,SP3_f1,SP4_f1, SP1_f2 |
| Hepg2 | MAZ_f1, SP4_f1, SP3_f1, SP1_f2, ZBT7B_si |
| Hmec | MAZ_f1, SP3_f1, SP1_f2, SP4_f1, ZBT7B_si |
| Hsmm | MAZ_f1, SP3_f1, IRF4_si, SP4_f1, SP1_f2 |
| Hsmmt | MAZ_f1, SP4_f1, SP3_f1, SP1_f2, ZBT7B_si |
| Huvec | FOXJ3_si, SP4_f1, PURA_f1, ZN148_si, RREB1_si |
| K562 | MAZ_f1, SP1_f2, SP3_f1, ZBT7B_si, IRF4_si |
| MonoCd14 | MAZ_f1, SP3_f1, SP1_f2, SP4_f1, ZBT7B_si |
| Nha | MAZ_f1, SP3_f1, ZBT7B_si, SP1_f2, SP4_f1 |
| Nhdfad | MAZ_f1, SP3_f1, SP1_f2, SP4_f1, IRF4_si |
| Nhek | MAZ_f1, SP3_f1, SP4_f1, SP1_f2, ZBT7B_si |
| Nhlf | MAZ_f1, IRF4_si , SP3_f1, SP4_f1,SP1_f2 |
